# Simulation of the ductile machining mode of silicon

**DOI:** 10.1007/s00170-021-07167-3

**Published:** 2021-05-14

**Authors:** Hagen Klippel, Stefan Süssmaier, Matthias Röthlin, Mohamadreza Afrasiabi, Uygar Pala, Konrad Wegener

**Affiliations:** 1 Department of Mechanical Engineering, Institute of Machine Tools and Manufacturing (IWF), ETH Zürich, Zürich, Switzerland; 2 Operation Center 1 at Federal Office of Meteorology & Climatology, MeteoSwiss, Switzerland; 3 Inspire AG, Zürich, Switzerland

**Keywords:** Grinding, Silicon, Machining, Diamond wire saw, High-performance computing, Particle simulation, SPH, GPGPU

## Abstract

Diamond wire sawing has been developed to reduce the cutting loss when cutting silicon wafers from ingots. The surface of silicon solar cells must be flawless in order to achieve the highest possible efficiency. However, the surface is damaged during sawing. The extent of the damage depends primarily on the material removal mode. Under certain conditions, the generally brittle material can be machined in ductile mode, whereby considerably fewer cracks occur in the surface than with brittle material removal. In the presented paper, a numerical model is developed in order to support the optimisation of the machining process regarding the transition between ductile and brittle material removal. The simulations are performed with an GPU-accelerated in-house developed code using mesh-free methods which easily handle large deformations while classic methods like FEM would require intensive remeshing. The Johnson-Cook flow stress model is implemented and used to evaluate the applicability of a model for ductile material behaviour in the transition zone between ductile and brittle removal mode. The simulation results are compared with results obtained from single grain scratch experiments using a real, non-idealised grain geometry as present in the diamond wire sawing process.

## Introduction

**Fig. 1:**
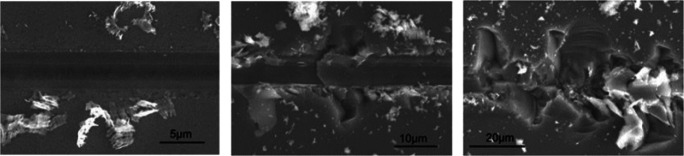
Diamond scribing of monocrystalline silicon: surface morphologies resulting from different depth of cut *h*_*cu*_: ductile removal *h*_*cu*_ = 0.123*μm* (left), transition from ductile to brittle removal *h*_*cu*_ = 0.722*μm* (middle), and brittle removal *h*_*cu*_ = 1.225*μm* (right) [2]

Hard and brittle materials, such as silicon, are difficult to machine as they exhibit high hardness and withstand high temperatures but show low resistance in shear and tension. In cutting operations, the material removal can change from ductile to brittle when increasing the depth of cut. Brittle cutting allows for higher material removal rates but leads to surface damage which is unwanted in the manufacturing process of silicon wafers and has to be removed by subsequent etching processes. Therefore, it is desired to generate the wafer surface in the ductile mode only. Figure 1 shows resulting surface morphologies when cutting with different cut depths where at a low cut depth, no surface damage occurs but at higher cut depths, larger surface damage and breakouts are visible. Silicon wafers are nowadays most efficiently produced by means of diamond wire sawing (DWS) [1], where geometrically undefined diamond grains fixed on a thin steel wire are indented into and moved across the workpiece material. Models used to describe the material removal process are typically built upon idealised geometries, leading to limitations when transferring results to the real process. Numerical simulations can help not only to understand phenomena associated with material removal processes but also allow to optimise the cutting process parameters such that a high material removal rate with acceptable surface properties can be achieved. Key problems are the modelling of the material behaviour as well as the numerical method to resolve the continuum.

This paper presents a simulation of silicon cutting with a single diamond grain. Its geometry was determined before cutting using optical microscopy. The resulting grain geometry was 3D meshed and used to drive a process simulation with numerical methods. Here, meshfree methods, in particular the smoothed particle hydrodynamics (SPH), were applied as they offer advantages in terms of computation times compared to classical mesh-based approaches: Upon large deformations, the continuum needs to be remeshed regularly, while meshfree methods automatically adapt to changes in the continuum. The goal of the study is to evaluate the applicability of the Johnson-Cook flow stress model to the cutting process of silicon in the transition zone between ductile and brittle material removal mode. Theoretical aspects of the SPH and cutting of silicon are introduced hereafter.

### SPH

SPH was introduced 1977 in astrophysics for the calculation of a smoothed density from point clouds [3]. A simple derivation of the method is based on the partition of unity [4], where a field value at a spatial location *x* can be determined as:

1 $$ f(x) = {\int}_{\mathbb{R}^{d}} \delta(x-x^{\prime})f(x^{\prime})d{\Omega}_{x^{\prime}} \forall x \in \mathbb{R}^{d}  $$

The Dirac-delta function *δ*(*x*) in Eq. 1 has two important properties:

2 $$ {\int}_{-\infty}^{+\infty} \delta(x)dx=1  $$

and

3 $$ {\int}_{-\infty}^{+\infty} \delta(\zeta-x)f(\zeta)d\zeta= f(x)  $$

Replacing the Dirac-delta function *δ* with a smoothing function, the so-called Kernel, *W*(*x* − *x*,*h*), e.g. the Gauß-function, with *h* being a smoothing length, the behaviour of the Dirac-delta can be reproduced for the limit:

4 $$ \lim_{h \rightarrow 0} W(x-x^{\prime},h)=\delta(x-x^{\prime})  $$

Inserting (4)–(3) gives an approximation of the function value f(x) at x:

5 $$ <f(x)> = {\int}_{-\infty}^{+\infty} W(x-x^{\prime},h)f(x^{\prime})dx^{\prime}  $$

This can be integrated within a discrete neighborhood using a Riemann-sum:

6 $$ <f_{i}> = \sum\limits_{j} f_{j} W(x_{ij},h){\Delta} V_{j}  $$

with the point index *i* at which the function value is to be approximated by its neighbor points *j*, *x*_*ij*_ is the spatial distance between point *i* and *j* and Δ*V*_*j*_ being an integration weight of point *j*. Computation of the function’s derivative leads to

7 $$ <\nabla f_{i}> = \sum\limits_{j} f_{j} \nabla W(x_{ij},h){\Delta} V_{j}  $$

where only the derivative of the Kernel *W*(*x*_*ij*_,*h*) is required. In this way, derivatives of values given at point cloud locations can be computed without the requirement of a functional description or a mesh-based relation between these points (particles). With this meshfree approximation derivatives in continuum mechanics equation can be computed by sums of discrete values in the particles neighborhood. Meshfree techniques were adopted in early 1990s to structural simulations [5] and for numerical cutting simulations first by [6]. At IWF of ETH Zürich the software *mfree_iwf* was developed in the past years for SPH based cutting simulations [7]. The software is capable of performing CPU as well as GPU-enhanced computations of metal cutting simulations. It is establishing the state-of-the-art in SPH simulations as it facilitates the most recent correctors and stabilisation measures for mechanical [8] and thermal simulations [9]. The software successfully demonstrated computationally highly efficient metal cutting simulations [10], identification of friction parameters [11], and its ability to adaptive refinements [12]. Furthermore, single grain grinding simulations of engineered grinding tools [13] were successfully demonstrated.

In the presented investigation, the software is adopted to simulate the cutting of silicon with a single diamond grain assuming isotropic behaviour of silicon with the Young’s modulus of Silicon in the 〈100〉 directions [14]. A flow stress model according to Johnson and Cook [15] came to application. The model is commonly used to describe metal plasticity within machining simulations and is given as:

8 $$ \begin{array}{ll} \sigma_{y} = & \left( A+B \cdot (\varepsilon_{pl})^{n}\right) \left( 1+C \cdot ln\left( \frac{\dot\varepsilon_{pl}}{\dot\varepsilon^{0}_{pl}}\right) \right) \cdot\\ & \left( 1- \left( \frac{T-T_{ref}}{T_{f}-T_{ref}} \right)^{m} \right) \end{array} $$

with *A*, *B*, *C*, *m*, and *n* being material parameters, *ε*_*pl*_ the current plastic strain, $\dot \varepsilon _{pl}$ the current plastic strain rate, and *T* the current temperature. *T*_*f*_ is the melting temperature, *T*_*ref*_ is the reference temperature, and $\dot \varepsilon _{pl}^{0}$ the reference plastic strain rate. The first two terms describe hardening due to plastic strain and plastic strain rate, respectively. The third term controls thermal softening upon increasing temperature. In this investigation, material parameters for silicon according to [16, 17] were used.

### Machining of silicon

Important contributions to the understanding of cutting of hard and brittle materials and the damage associated were first introduced by Hamilton and Goodman [18] and Lawn [19] who presented analytical models of the pressure field under a sliding indenter. The mechanics and mechanisms of plastic deformation and fracture of brittle materials upon indentation have been studied by Lawn et al. They introduced the median-radial crack system [20], as well as the lateral crack system [21]. Bifano et al. introduced the possibility of machining brittle materials in ductile cutting regime [22] and presented a model describing the critical depth of cut *h*_*cu*,*crit*_ which presents a threshold above which brittle fracture is introduced. Based on these concepts, the ductile machining of silicon has been extensively studied taking into account the effects of various aspects. Larger cutting edge radii lead to higher normal and cutting forces [23], increase the critical depth of cut [24], and lead to horseshoe-like cracks in the grooves rather than radial-chevron cracks [25]. The critical depth of cut is very sensitive to the crystallographic plane and direction and varies between 0.112*μm* in (001)[010] and 1.270*μm* in $(110)[1\bar {1}\bar {1}]$ [26]. High cutting speed, as present in DWS of silicon, leads to amorphisation of silicon which is accompanied by a volumetric expansion and consequently lower residual cutting depth [27]. Furthermore, phase transitions happen due to high contact pressure [28–30], which affect the mechanical properties of the material and therefore the material removal behaviour. Recently, modelling the elastic stress field has gained attention again to predict damage [31–33] with good success in predicting the direction of cracks, their frequency, and their length.

With exception of Bifano’s work, all studies used idealised grain shapes to study the respective phenomena, presenting a limitation to their applicability towards grinding processes. In order to exploit the freedom of geometry presented by the SPH method, the experiments conducted in this study use a non-idealised grain shape.

## Experimental methodology and results

The cutting tests were performed with one single grain from a diamond wire used for the diamond wire sawing process of silicon wafers. The tests were conducted for different depths of cut *h*_*cu*_ and the process forces *F*_*N*_ and *F*_*C*_ as well as the resulting surface properties were recorded.

A summary is presented in the following sub-chapters, along with a discussion of experimental results.

### Optical measurement of the grain geometry

**Fig. 2:**
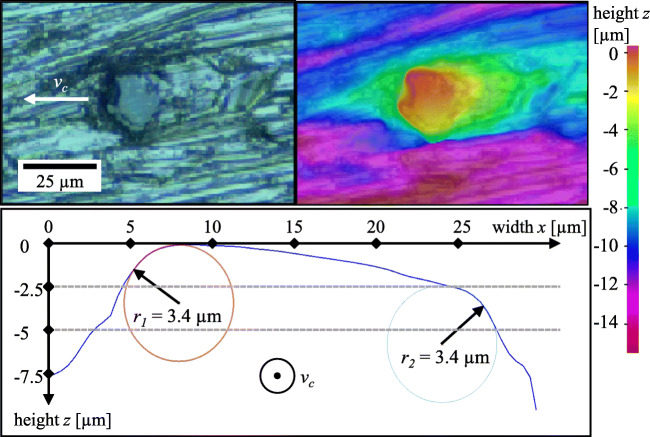
The diamond grain used for the experiments and simulation shows approximately 10*μm* protrusion and a width of 30*μm*. Top left: microscopic image with indication of the cutting direction; top right: false colour image of the grain showing the height profile; bottom: cross-sectional view of the grain projected into the cutting direction

The grain chosen for the scratch test is shown in Fig. 2. The grain is embedded on a commercial diamond wire; the metallic matrix covering it has been carefully removed with sand paper. The wire is bent over a pin in such a way that the selected grain is isolated on the highest point.

**Fig. 3:**
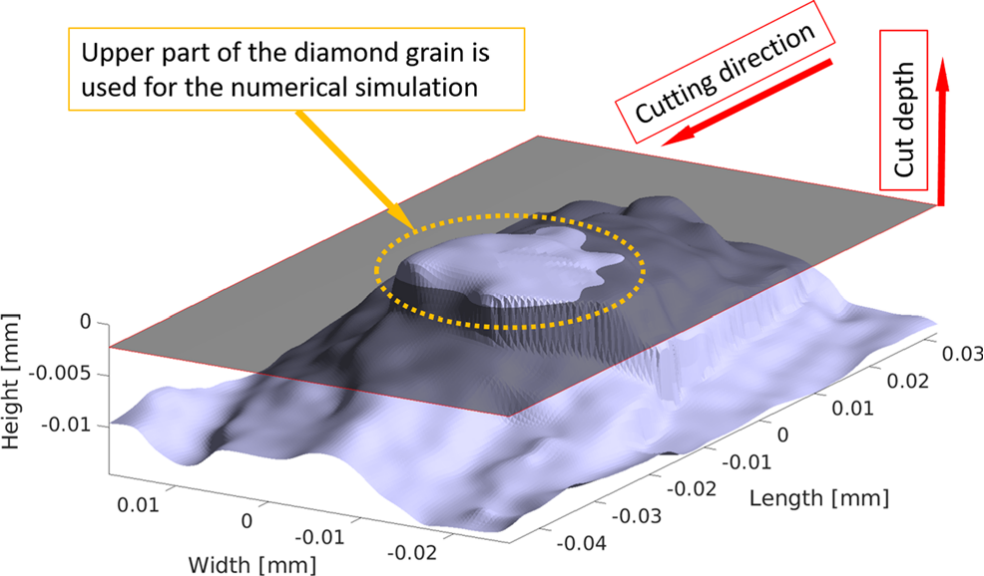
Triangulated surface of the point cloud from 3D microscope measurement. Above the black plane, the relevant model portion which was later considered for the numerical cutting simulations is indicated

Prior to the scratch test, this diamond grain was optically measured with an Alicona microscope and the surface data was retrieved as a point cloud. This point cloud was triangulated in MATLAB and a 3D mesh with tetrahedrons was created from the top part of the diamond as the cutting takes place in this region only. The surface mesh is shown in Fig. 3, the geometry part above the black plane indicates the relevant part for the simulations conducted.

Due to the geometric properties of fractured diamond grains used on electroplated diamond wires, only some of the parameters typically used to describe the tool geometry in micro machining can be applied: The projected cross-section of the grain is approximately trapezoidal with a slight tilt of the flat as shown in Fig. 2. The radii at the grain flanks are approximately 3*μm* on both sides, while the left side in direction of cut (right side in Fig. 2) is only engaged at large depth of cut of approximately 3*μm*. The cutting edge radius varies between 1.4 and 6.7 *μm* over the cutting edge and is smallest at the sides and larger in the centre of the grain. Rounding of the edge due to wear leads to an increase with increasing scratch length. The width-to-height ratio *r* of the grain is evaluated for each simulated depth of cut *h*_*cu*_ separately: $r_{h_{cu}=0.2 \mu m} \approx 27$, $r_{h_{cu}=0.5 \mu m} \approx 17$, $r_{h_{cu}=1 \mu m} \approx 13$, and $r_{h_{cu}=1.5 \mu m} \approx 11$.

### Cutting Experiments

**Fig. 4:**
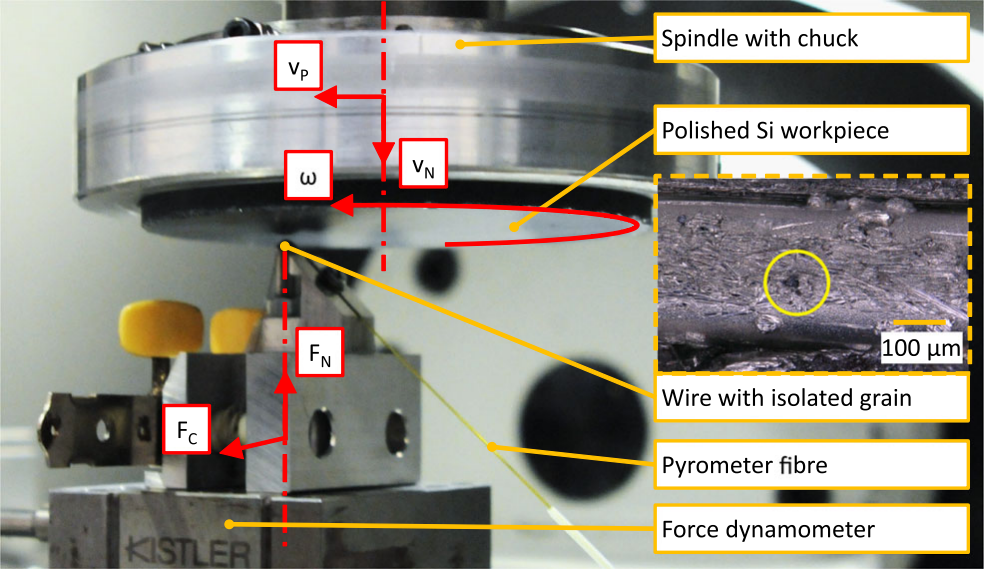
Experimental set-up, indicating relevant components and kinetic and kinematic quantities

The cutting tests were performed on a Fehlmann Picomax Versa 825 5-axis milling machine. The set-up is shown in Fig. 4.

A polished silicon surface is attached to and rotated with the spindle with angular velocity *ω*. The diamond grain on the wire is isolated by bending it over a pin and mounted onto a three-component force dynamometer (Kistler 9256C, eigenfrequency *f* ≈ 6*kHz*) fixed to the stationary machine table. The force measurement set-up is completed by a Kistler Type 5080A charge amplifier with active drift compensation and 1000-Hz low-pass filter and a National Instruments NI 9222 A/D converter, sampling each channel at 211.64*kS*/*s*. A pyrometer fibre was furthermore installed and connected to a Fire-3 two-colour fibre optic pyrometer for the measurement of flash temperatures. The diamond grain is kept stationary while the rotating spindle is fed vertically into the grain with normal feed speed *v*_*f*_ and horizontally across the grain with lateral feed speed *v*_*p*_ in such a way that the vertical displacement is equal to 3*μm* and the horizontal displacement is equal to 1000*μm* over 20 spindle rotations. The angular velocity *ω* is chosen in such a way that the resulting cutting speed *v*_*c*_ equals 10*m*/*s*. The motion results in spiral-shaped contact path; due to a small but relevant perpendicularity error of the Si surface with the spindle axis, the cut is interrupted and the engagement length equals roughly 4*cm* per revolution.

**Fig. 5:**
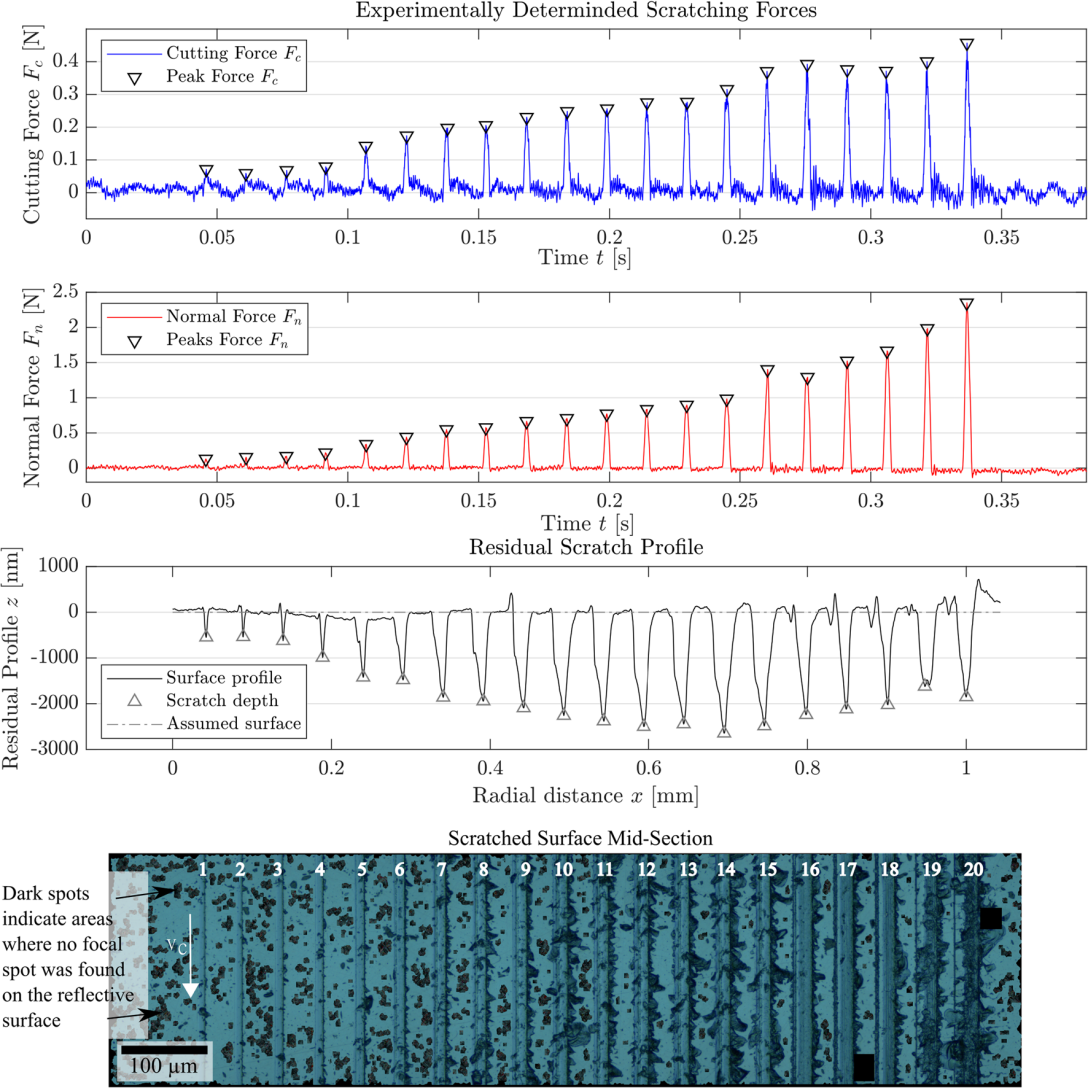
Experimentally captured forces and surface profile (median depth evaluated in the depicted area orthogonal do the depth of cut) aligned with a microscopic image of the surface. Scratch direction top to bottom; vertical infeed increasing from left to right. Smooth sections as well as brittle fracture, especially on the sides of the scratch, are well visible

The topography of the scratches is optically evaluated at their centre, where the engaged depth is largest. The residual surface aligned with the resulting topography profile line is shown in the bottom of Fig. 5. The residual profile is generated by plotting the median profile height for all data points that lie on a line in cutting direction at a given radial distance *x*. The median value has proven to be closer to the profile depth of a manually chosen representative section of each scratch than the arithmetic mean.

The residual scratch depth *h*_*res*_ for each pass is identified as the vertical distance from a reference height determined in the unscratched area of the workpiece, shown as a dashed line “assumed surface” in Fig. 5. Cutting force *F*_*c*_ and normal force *F*_*n*_ are identified from the peaks in the respective signals and also aligned with the surface topography. All experimentally determined values are compiled in Table 1.

**Table 1:** Experimental data acquired from force and topography measurement; scratch numbers refer to the enumeration in Fig. 5

Scratch	Removal	Resid. depth	Norm. force	Cut. force
#	mode	*h*_*res*_ [*μm*]	*F*_*N*_ [N]	*F*_*C*_ [N]
1	Ductile	0.55	0.129	− 0.071
2	Ductile	0.54	0.153	− 0.059
3	Ductile	0.62	0.168	− 0.068
4	Ductile	0.99	0.218	− 0.079
5	Ductile	1.42	0.337	− 0.142
6	Ductile	1.48	0.440	− 0.174
7	Transition	1.86	0.548	− 0.197
8	Transition	1.94	0.575	− 0.205
9	Transition	2.09	0.665	− 0.230
10	Brittle	2.26	0.706	− 0.248
11	Brittle	2.38	0.771	− 0.256
12	Brittle	2.50	0.835	− 0.275
13	Brittle	2.44	0.896	− 0.277
14	Brittle	2.65	0.984	− 0.315
15	Brittle	2.49	1.402	− 0.370
16	Transition	2.24	1.292	− 0.392
17	Transition	2.19	1.523	− 0.376
18	Transition	2.02	1.666	− 0.371
19	Brittle	1.62	1.985	− 0.400
20	Brittle	1.85	2.350	− 0.457

### Discussion of experimental results

The determination whether the removal mode is mainly ductile, brittle, or in the transition is subjective and based on the optical appearance of the scratch. Appearance of cracks and pittings in the bottom of scratch is used as a transition criterion; however, also scratches classified as ductile show some fracture on the side.

Cracks on the side of the scratch appear first on the side with the larger engagement (left side in the bottom of Fig. 2). Their appearance is furthermore dominant in the regions of the grain where the cutting edge radius is small in agreement with [24].

It is noteworthy that scratches with large proportions of material removed in ductile cutting mode are observed with residual depths much larger than the critical depth of cut proposed by Bifano et al. and determined to be in the vicinity of 60*nm* for silicon [34], that is, in spite of elastic recovery which happens after the cut and renders the engaged depth deeper than the residual depth. The observation is, however, in agreement with findings published in [26], where the critical depth of cut for ductile removal of silicon with a cone shaped indenter with a cutting edge radius of 8*μm* was determined to lay between 0.122 − 1.270*μm* depending on the crystallographic plane and orientation.

It is furthermore observed, that the scratches 16–18 show a ductile bottom of the scratch with comparably few breakouts on the edges while the scratches before and especially the scratches after them show significant brittle fracture also on the bottom of the scratch. The residual scratch depth decreases over the last scratches in spite of the increasing in-feed (evident in the increasing normal force), which might be attributed to increased amorphisation and associated volumetric expansion [27] and increased elastic recovery.

It was further attempted to quantify the elastic recovery and the undeformed chip thickness from the experimentally determined residual depths by applying models found in literature. For ductile removal, a reasonable undeformed chip thicknesses that can be used in this context could not be determined, which is mainly due to the large deviations between the idealised indenter geometries used to develop the model and the real grain geometry used in this study. The model has to be developed further which goes beyond the scope of this study. For this reason, comparison between experimental and stimulative results is based on residual scratch depth only. The models evaluated are only described briefly and can be referred to in the references mentioned.

For ductile material removal, indentation theory proposed by Oliver and Pharr [35] and a model developed and validated for single grain scratching of silicon by Huang et al. [36] is applied to consider elastic recovery, with the assumption that the grain tip is spherical. For a spherical indenter, the actively engaged tip radius can be determined from the residual scratch width and the contact depth. With a known tip radius and the assumption that the residual scratch width is equal to the width of the tool tip in engagement [35], it is possible to solve the equations for the undeformed chip thickness .

In case of brittle material removal, a model developed by Marshall et al. [21] can be applied to accommodate spalling of material due to brittle fracture. Since both the scratch width and depth are affected by brittle removal, consideration of forces and material parameters is required to determine the undeformed chip thickness. The determination requires the assumption that the depth of the material removed equals the depth of the plastic zone [21] and that material on the side of the groove is removed due to the propagation of lateral cracks to the surface [37]. The plastic zone extends equally in half width and depth and equals the residual, measured depth of the scratch, while the length of the lateral cracks equals half of the measured width of the residual scratch. The engaged depth equals the undeformed chip thickness [21]. Details of the application of this approach to real grain geometries can be found in [38].

## Numerical model

**Fig. 6:**
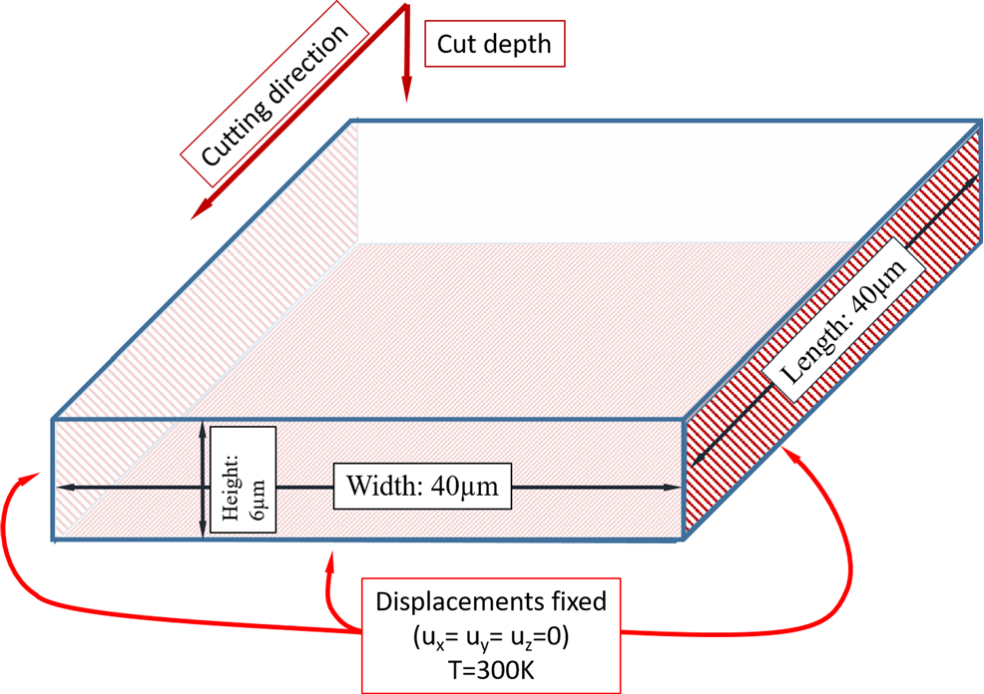
Workpiece dimensions and boundary conditions

The modelled workpiece geometry has a size of 40*μm* by 40*μm* by 6*μm* (length, width, and height) and is discretised with 259^′^200 (120×120×18) particles. The workpiece is restrained at the bottom, left, and right faces and a temperature boundary condition of *T* = 300*K* is applied. Geometry and boundary conditions are depicted in Fig. 6.

**Fig. 7:**
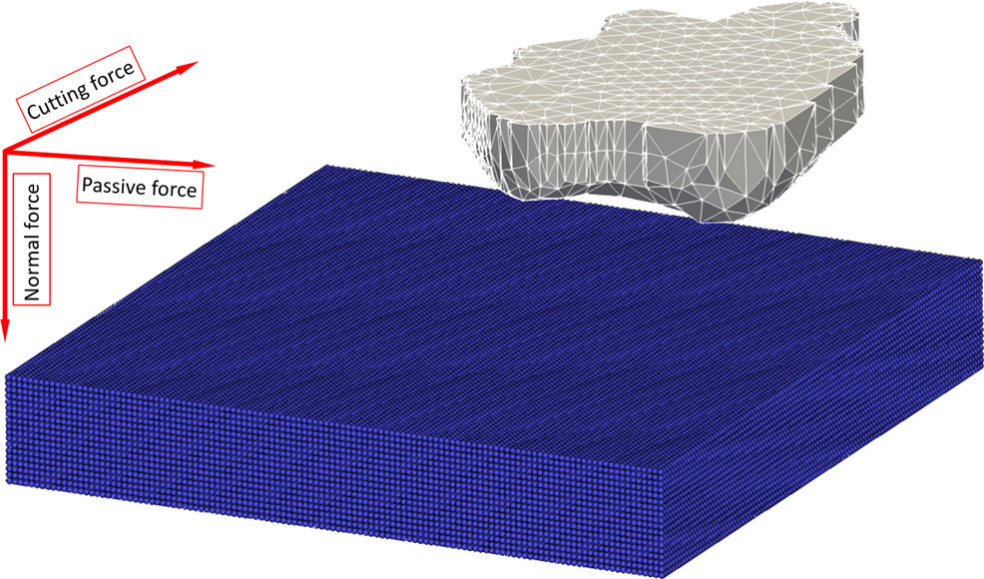
Workpiece discretisation and meshed single grain diamond

The diamond grain is modelled as a rigid body with a mesh consisting of 4426 tetrahedron elements. The discretised workpiece and meshed diamond grain are shown in Fig. 7 together with the directions of the acting process forces. Heat conduction is considered in the workpiece using the Particle Strength Exchange (PSE) approximation of the Laplacian. Tool heat transfer was not considered.

Coulomb friction is used with a friction coefficient assumed to be *μ* = 0.3. The fraction of plastic work that is converted into heat (Taylor-Quinney coefficient) in silicon cutting is unknown and is assumed here to be *η*_*TQ*_ = 90*%* for all simulations. Heat generation due to friction is considered with $\eta _{fric}^{WP} = 8.5\%$ of the dissipated frictional energy being transferred into the workpiece (WP). The ratio stems from the assumption of a proportional split of the frictional heat transferred into tool and workpiece according to their respective thermal diffusivities *α*_*WP*_ and *α*_*Dia*_. This thermal diffusivity ratio between workpiece (silicon) and tool (diamond^1^) is computed with:

9 $$  \eta_{fric}^{WP} = \frac{\alpha_{WP}}{\alpha_{Tool}+\alpha_{WP}} = \frac{\frac{\lambda_{WP}}{{\varrho}_{WP}\cdot cp_{WP}}}{\frac{\lambda_{Dia}}{{\varrho}_{Dia}\cdot cp_{Dia}}+\frac{\lambda_{WP}}{{\varrho}_{WP}\cdot cp_{WP}}} = 0.085 $$

**Fig. 8:**
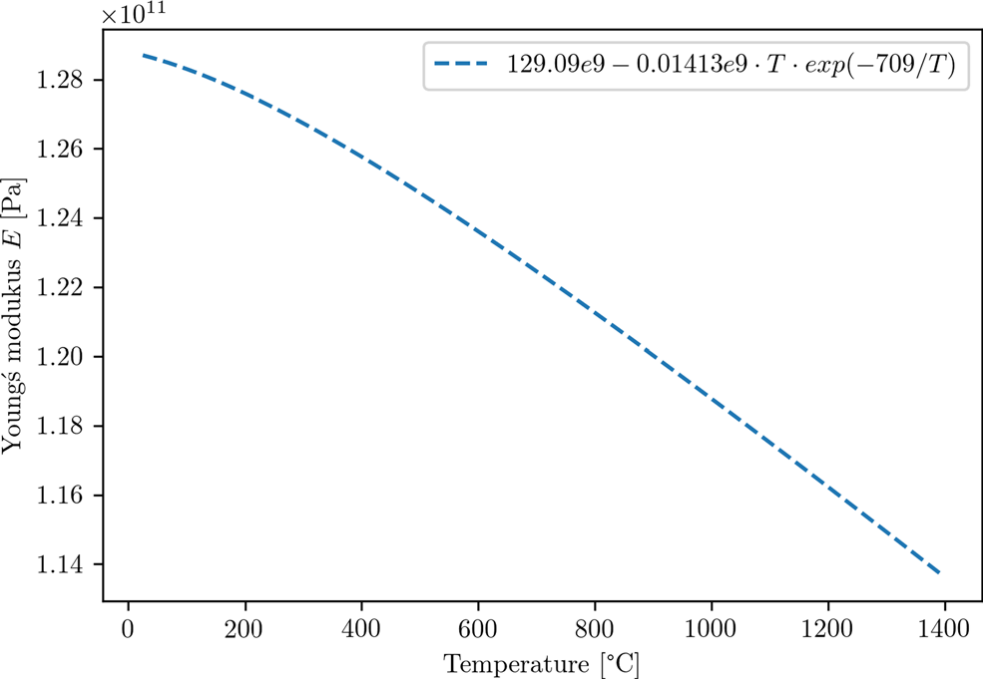
Temperature dependent Young’s modulus *E*< 100>[14] used in this investigation

**Fig. 9:**
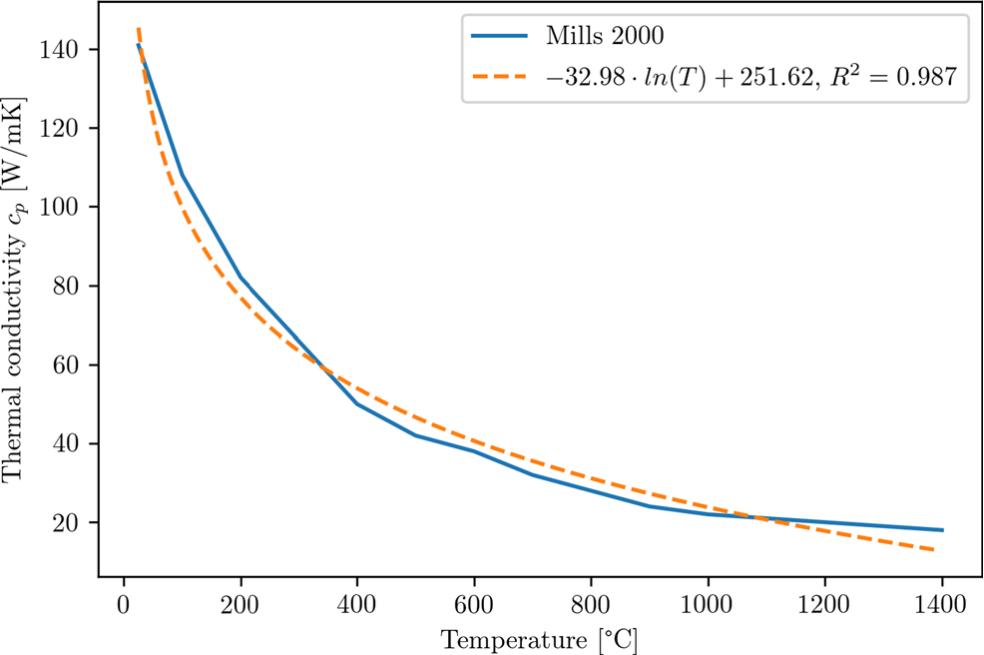
Temperature dependent thermal conductivity [39] and approximation used in this investigation

**Fig. 10:**
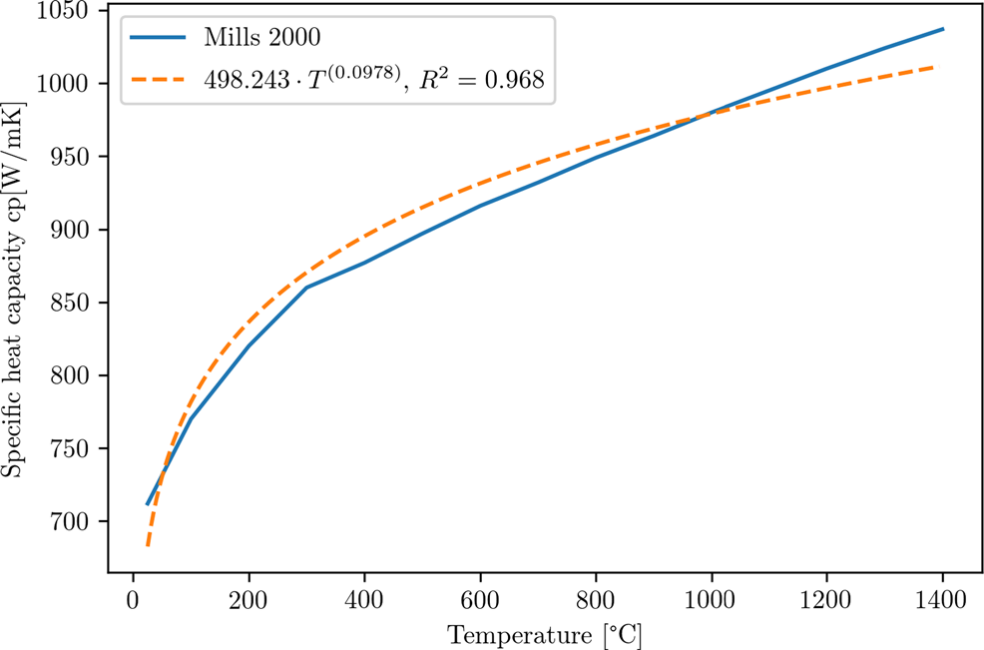
Temperature dependent specific heat [39] and approximation used in this investigation

Temperature-dependent properties for the Young’s modulus (Fig. 8), the thermal conductivity (Fig. 9), and the specific heat (Fig. 10) are used. All material parameters used in the simulations are summarised in Table 2.

**Table 2:** Material parameters for silicon used in the simulation

Parameter	Symbol	Value	Unit	Data Source / Comments
Young’s modulus *E*〈100〉	*E*(*T*)	129.09*e*9 − 0.01413*e*9 ⋅ *T* ⋅ *exp*(− 709/*T*)	Pa	[14], see also Fig. 8
Poisson ratio	*ν*	0.273	-	[16]
Density	*ϱ*	2328	kg/m^3^	[16]
Specific heat	*c*_*p*_(*T*)	498.243 ⋅ (*T* + 273*K*)^(0.0978)^	$\frac {J}{kgK}$	Power law fit to data from [39], see Fig. 10
Thermal conductivity	*λ*(*T*)	− 32.98 ⋅ *ln*(*T* + 273*K*) + 251.62	$\frac {W}{mK}$	Logarithmic function fit to data from [39], see Fig. 9
Friction coefficient	*μ*	0.3	-	Author’s assumption
Fraction of friction energy released as heat into the workpiece	$\eta _{fric}^{WP}$	0.085	-	Thermal diffusivity ratio silicon/diamond, see Eq. 9
Taylor-Quinney coefficient	*η*_*TQ*_	0.9	-	Author’s assumption
JC constant	A	896.394	MPa	[16]
JC constant	B	529.273	MPa	[16]
JC constant	C	0.4242	-	[16]
JC constant	m	1	-	[16]
JC constant	n	0.3758	-	[16]
JC constant	$\dot \varepsilon _{pl}^{0}$	1	1/s	[16]
Melting temperature	*T*_*f*_	1688.15	K	[16]
Reference temperature	*T*_*ref*_	300.0	K	Author’s assumption

The simulations are performed for cut depths *h*_*cu*_ of 200*nm*, 500*nm*, 1000*nm*, and 1500*nm* and a cutting speed of *v*_*c*_ = 10*m*/*s* as in the conducted cutting experiments. An overview of all simulated process parameters is provided with Table 3, along with the resulting forces. All simulations are carried out with GPU acceleration on a NVIDIA Quadro GP100 graphics card. The computation time of each scratch simulation was in the order of 5.75 h for $\thicksim 270'000$ time steps.

**Table 3:** Overview of simulated process parameters and resulting forces, temperatures, and residual depths

Simulation	Cutting speed	Depth of cut	Cutting force	Normal force	Max. Temperature	Residual depth
#	*v*_*c*_ [m/s]	*h*_*cu*_ [nm]	*F*_*C*_ [N]	*F*_*N*_ [N]	*T*_*max*_ [K]	*h*_*res*_ [nm]
1	10	200	-0.009	0.030	310	10
2	10	500	-0.071	0.196	1100	100
3	10	1000	-0.121	0.295	1700	800
4	10	1500	-0.152	0.322	1700	1300

## Results and discussion

Results of the simulations are discussed in comparison with the experiments conducted and literature findings

### Surface topography and elastic recovery

**Fig. 11:**
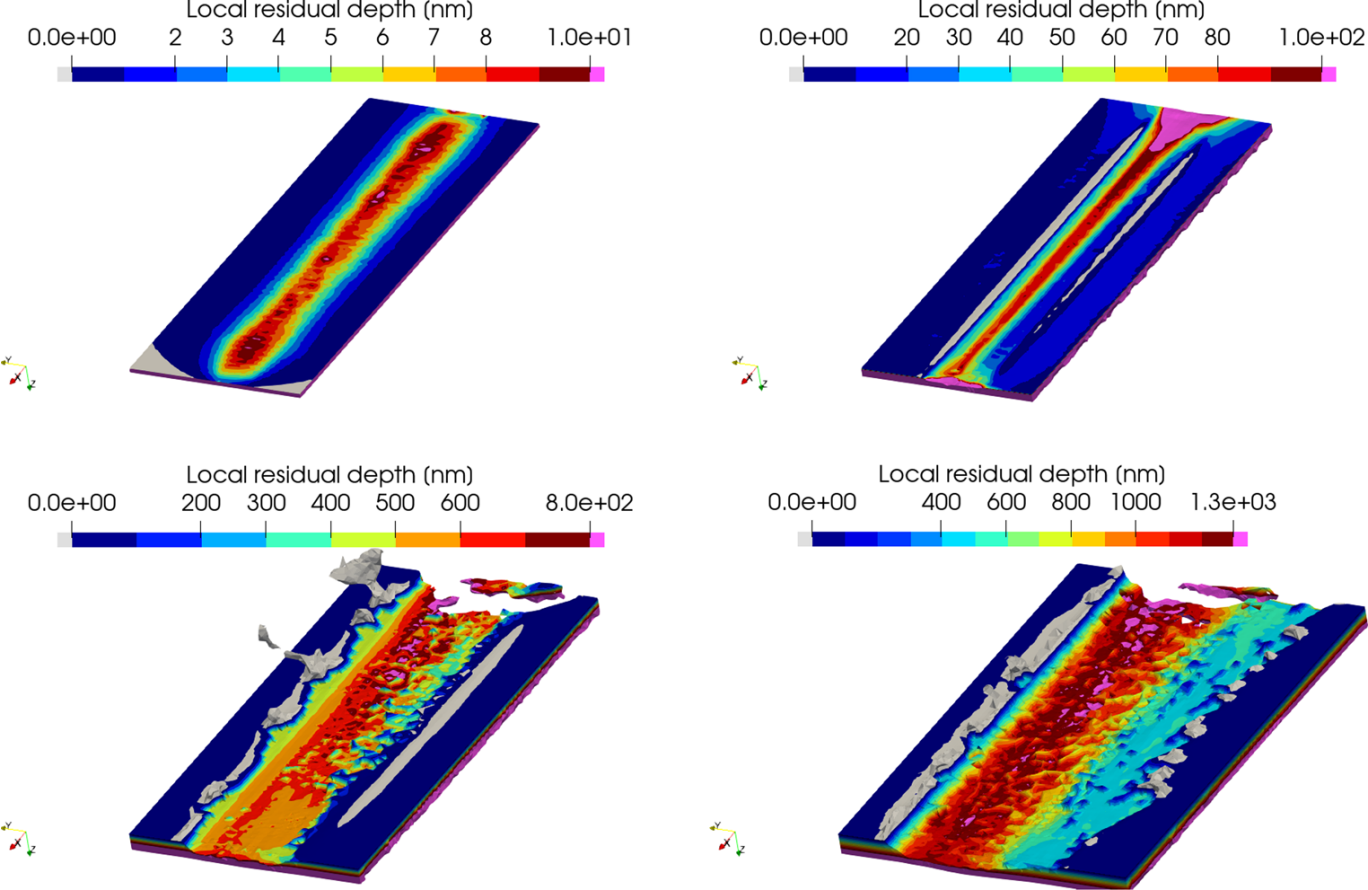
Local residual depths [nm] for the simulation with *h*_*cu*_ = 200*nm* (top left), *h*_*cu*_ = 500*nm* (top right), *h*_*cu*_ = 1000*nm* (bottom left) and *h*_*cu*_ = 1500*nm* (bottom right). The cutting direction is from bottom to top. Note that different colour scales are used. Dark-blue-coloured regions are related to material which is still in the initial zero-plane of the surface, while material accumulation and debris extending above the initial surface are coloured in grey

For the generation of the residual profile and estimation of the elastic recovery, the SPH particle positions at the simulation end are triangulated with Open3D [40] and the depth coordinates are mapped from the particles to the mesh. Figure 11 shows the residual depth after the cut.

The numerical simulation predicts for the lowest depths of cut *h*_*cu*_ = 200*nm* and *h*_*cu*_ = 500*nm* no chip or debris formation, while for cut depths of *h*_*cu*_ = 1000*nm* and *h*_*cu*_ = 1500*nm*, a chip develops in front of the tool as well as smaller breakouts and debris sideways of the tool are created. These breakouts are much smaller than those observed in the experiments

**Fig. 12:**
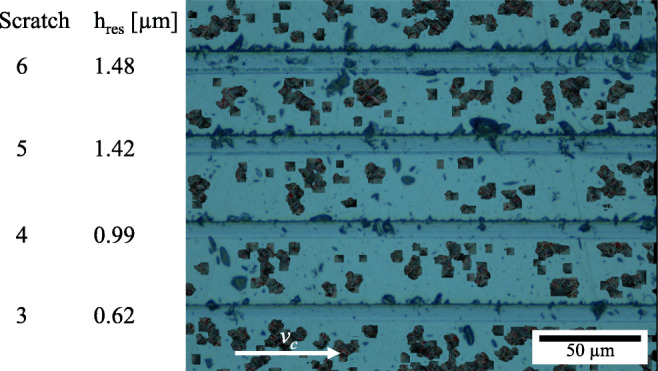
Enlarged view of the relevant experimentally generates scratches #3–6

The first two simulated scratches with *h*_*cu*_ = 200*nm* and *h*_*cu*_ = 500*nm* result in very shallow groves of *h*_*res*_ = 10*nm* and *h*_*res*_ = 100*nm* respectively which cannot be matched up with experimental results as the lowest experimentally determined residual depth is 550*nm* and therefore much larger. The 3rd and 4th simulated scratches produce groves of a residual depth of *h*_*res*_ = 800*nm* and *h*_*res*_ = 1300*nm* respectively, which lays in the vicinity of the experimental scratches number 3 to 6, for which an enlarged view is provided in Fig. 12.

The general appearance of the simulated scratches agrees with that of the experimentally cut ones: the left flank in direction of cut is steeper with “break-outs” which are significantly deeper than those on the right flank. The bottom of the grooves gets rougher with increasing residual depths in both cases.

For the lowest simulated depth of cut of *h*_*cu*_ = 200*nm*, the imprint has a maximum depth of around 10*nm* which implies an elastic recovery depth *h*_*rec*_ of 190*nm*. At *h*_*cu*_ = 500*nm*, the elastic recovery is around 400*nm* and for *h*_*cu*_ = 1000*nm* and *h*_*cu*_ = 1500*nm* about 200*nm*. In comparison with experimental results obtained from grinding of silicon with grains of similar average size (≈ 25.3*μm*) and cutting speed of *v*_*c*_ = 44*m*/*s* [36], the simulated recovery depth is higher for small depth of cut and lower for high depth of cut. Recovery depth of $h_{rec, h_{cu} = 200nm} \approx 100 nm$, $h_{rec, h_{cu} = 500nm} \approx 200 nm$, and $h_{rec, h_{cu} = 800nm} \approx 300 nm$ were observed in the grinding experiments.

### Process forces

**Fig. 13:**
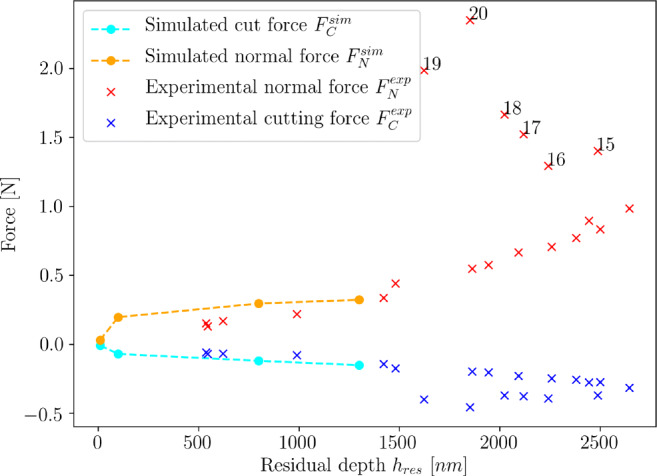
Measured and simulated cutting (*F*_*C*_) and normal (*F*_*N*_) forces versus cut depth *h*_*cu*_. The numbers with the outliers in the experimental normal force data points indicate the numbers of the respective scratch

In comparison to the experimental values from Table 1, the predicted process forces are generally higher. The errors in the prediction become smaller towards higher depth of cut *h*_*cu*_. A comparison of the experimentally and numerically determined forces is provided with Fig. 13 where forces are plotted over the residual depth *h*_*res*_.

While the numerical cutting force is in an acceptable agreement to the experimental values, the simulated normal force tends to be higher. The reason for the over-estimation of the normal forces may lie with the very low fracture toughness of silicon of < 1 MPa*m*^1/2^ [16] which is not considered with the modelling approach here. A small flaw in the workpiece initiates crack growth and brittle fracture with low energy intake. The cracks will not lead to breakouts as long as the hydrostatic stress is compressive; however, the residual hydrostatic stresses in the wake of the tool are partially in tension (refer to chapter Section 4.4) and indicate that the breakouts may likely occur. The ductile deformation and shearing of the material which are simulated require significantly more energy leading to higher forces since fracture is not modelled.

**Fig. 14:**
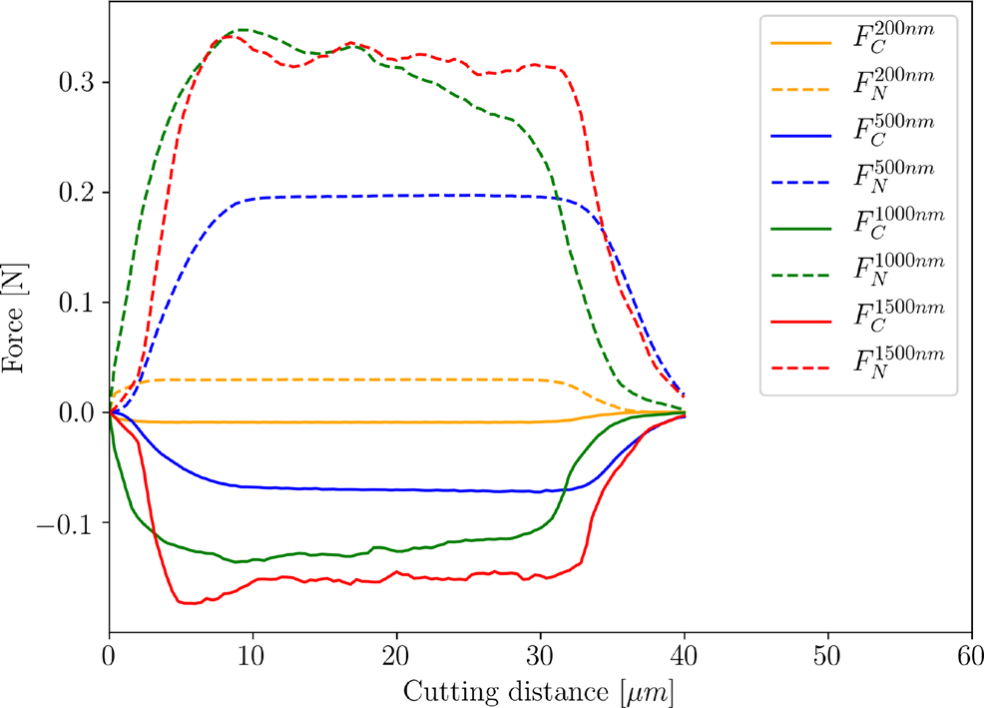
Predicted process forces at a cutting speed of *v*_*c*_ = 10*m*/*s* and depth of cut from *h*_*cu*_ = 200*nm* − 1500*nm*

The simulated cutting and normal force characteristics during the cut are shown for all simulations in Fig. 14. At increased depth of cut, the process forces are slightly varying due to formation of chips and small debris as well as torn out material.

### Hydrostatic pressure

**Fig. 15:**
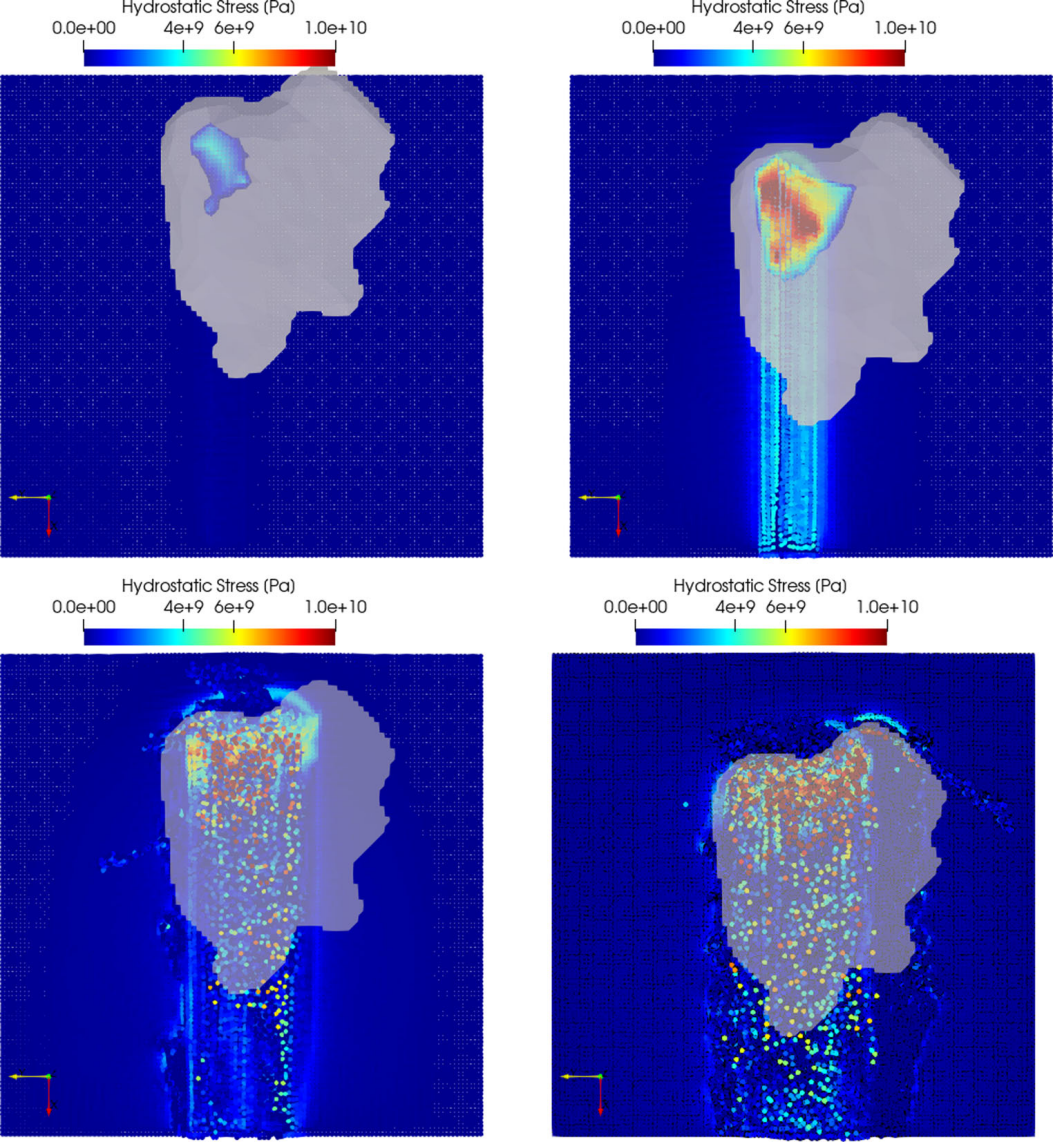
Hydrostatic pressure distribution below the cutting tool at cut depths of *h*_*cu*_ = 200*nm* (top left), *h*_*cu*_ = 500*nm* (top right), *h*_*cu*_ = 1000*nm* (bottom left), and *h*_*cu*_ = 1500*nm* (bottom right). The cut direction is from bottom to top

The hydrostatic pressure is investigated below the cutting tool where positive values are in compression. The pressure distributions are displayed in Fig. 15 with the tool shown transparent to visualise the hydrostatic pressure distribution below the tool as well. A video of this simulation is uploaded to https://youtu.be/nMTaoP0XIj4.

The increasing contact area with increasing depth of cut can be seen. The region of maximum hydrostatic pressure shifts towards the right side (in cutting direction) of the grain. At the lowest simulated depth of cut *h*_*cu*_ = 200*nm*, the maximum hydrostatic pressure is in the order 4GPa, at *h*_*cu*_ = 500*nm* increasing to 13 GPa while with further increasing cut depth the area of highly compressed zones increased steadily and surpassed in some spots even 60GPa. The levels of hydrostatic pressures found in the numerical prediction give rise to the assumption that phase transformation in the workpiece will occur, as for example described in [28] and simulated and validated in [27]. At the lowest cut depth, very small hydrostatic pressures in compression remain in the wake of the tool, while at *h*_*cu*_ = 500*nm* they are in the order of 3 − 4*GPa*. At both larger depths of cut of *h*_*cu*_ = 1000*nm* and *h*_*cu*_ = 1500*nm*, the residual hydrostatic pressures are lower in the cut surface compared to *h*_*cu*_ = 500*nm* but exceed 5 GPa locally (green to red in Fig. 15). On the other hand, they turn partially into tension (dark-blue in Fig. 15). This observation is in general agreement with a SPH simulation presented in [41] where cutting of KDP crystal was modelled by means of constitutive model based on the relative length of crack.

### Stress distribution

**Fig. 16:**
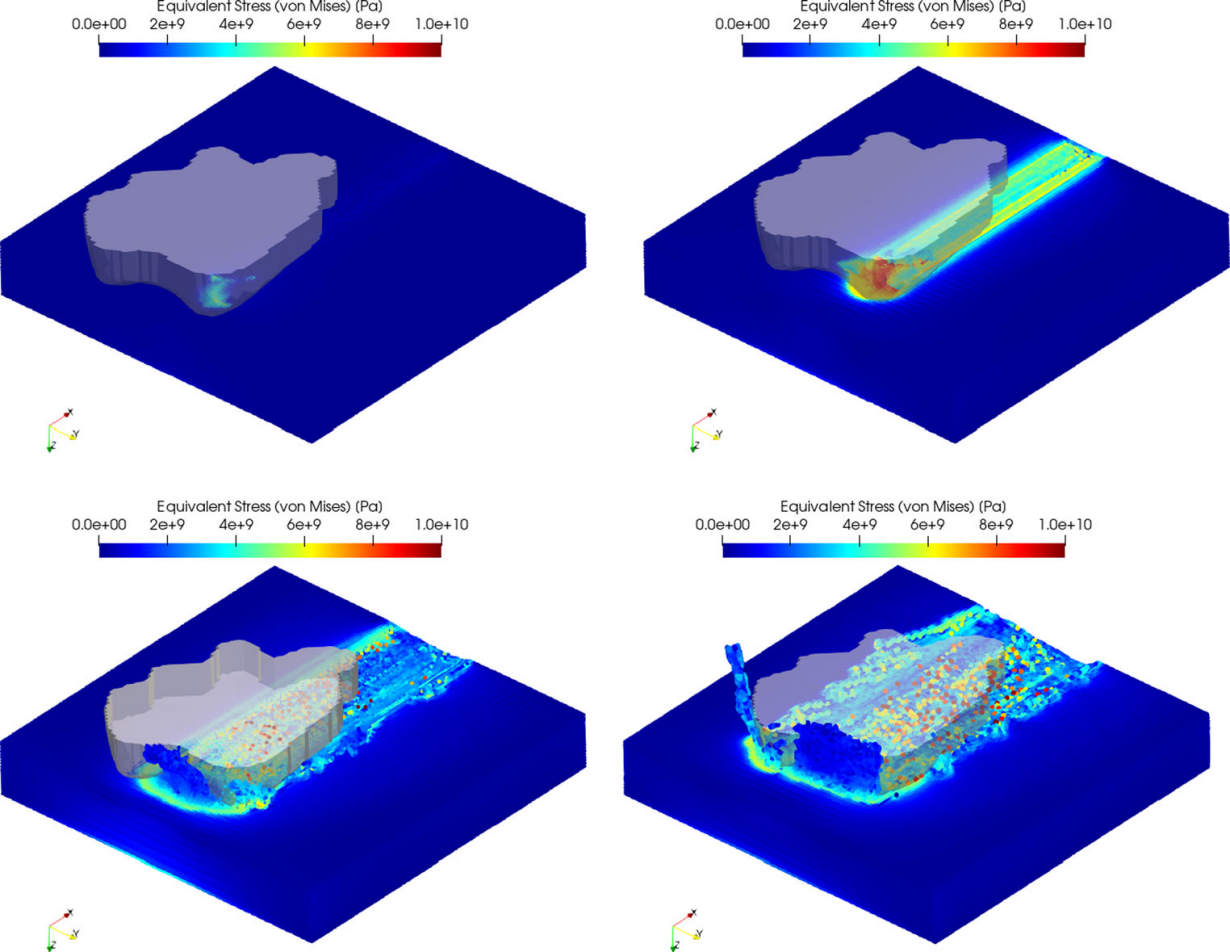
Equivalent stress distribution (von Mises) at cut depths of *h*_*cu*_ = 200*nm* (top left), *h*_*cu*_ = 500*nm* (top right), *h*_*cu*_ = 1000*nm* (bottom left), and *h*_*cu*_ = 1500*nm* (bottom right). The cut direction is from right to left

Figure 16 shows the stress distributions for the four depths of cut . The equivalent stress reaches up to 5.5 GPa under the tool at *h*_*cu*_ = 200*nm* and increases locally up to 10–11 GPa for the three higher depths of cut of *h*_*cu*_ = 500*nm*, *h*_*cu*_ = 1000*nm*, and *h*_*cu*_ = 1500*nm*. A video of the simulations is uploaded to https://youtu.be/QZw88fNM1CA.

In the wake of the tool, residual stresses form which, when overlayed with tensile hydrostatic pressures, potentially lead to opening of cracks and breakouts. The cracks are not accommodated for in the Johnson-Cook flow stress model but are visible the bottom of the experimentally generated scratches in Fig. 12.

### Predicted temperatures

**Fig. 17:**
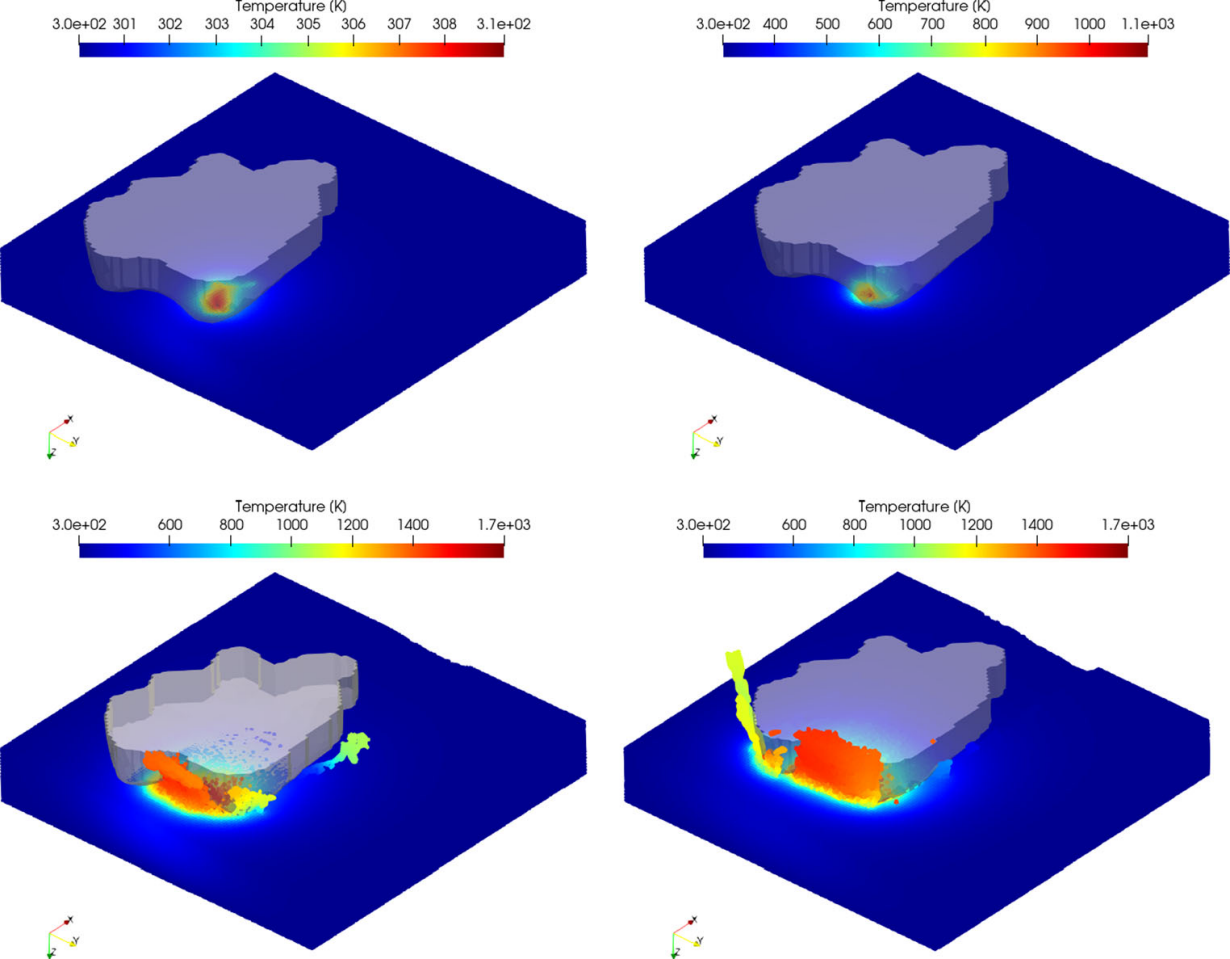
Temperature distribution at cut depths of *h*_*cu*_ = 200*nm* (top left), *h*_*cu*_ = 500*nm* (top right), *h*_*cu*_ = 1000*nm* (bottom left), and *h*_*cu*_ = 1500*nm* (bottom right). The cut direction is from right to left. Note that different temperature scales are used

The predicted peak temperatures in the workpiece strongly depend on the cut depth *h*_*cu*_ and increase from around *T* = 310*K* (*h*_*cu*_ = 200*nm*) up to the melting temperature *T* = 1700*K* for *h*_*cu*_ = 1000*nm* and *h*_*cu*_ = 1500*nm*, see Table 3 and Fig. 17. The temperatures at the higher depths of cut are in line with observations for experiments conducted in the same manner as the one presented in this study [38] where the flash temperature was measured to lie in the range around 1500*K*; however, the experimental results show that the temperature decreases with increasing depth of cut and brittle behaviour. At lower cut depths of *h*_*cu*_ = 200*nm* and *h*_*cu*_ = 500*nm*, the temperatures are therefore well below the experimental values. For this reason, the accuracy of the temperature prediction is questionable. A video of the simulations is uploaded to https://youtu.be/Vw4zPtsh3Dohttps://youtu.be/Vw4zPtsh3Do.

## Conclusion

Single grain cutting in silicon in ductile mode was simulated with the SPH and compared with experimental findings applying the same non-idealised grain geometry. As expected, the results indicate that the literature data for the Johnson-Cook flow stress model parameters have limited validity for depths of cut that lay in and above the ductile-to-brittle transition.

The process forces are predicted higher than experimentally observed, where the cutting forces only slightly exceed the experimental values which indicates that the assumed friction value close to the correct value. Since brittle fracture requires only a small amount of energy, it is concluded that the resulting tool forces are mainly induced by the ductility of the silicon.

While at the lowest simulated cutting depth compressive hydrostatic pressure reached around 4*GPa*, it then increased to values of 13*GPa* at a cutting depths of *h*_*cu*_ = 500*nm* which is sufficient for phase transformations in the silicon. Simulations with increased depth of cut showed strongly increased hydrostatic pressures under the cutting tool in excess of 60*GPa* locally. These high values at larger cutting depths have to be treated with care as the normal force predictions deviate from the experimental values and therefore are subject to changes upon improved constitutive modelling.

Predicting the transition from ductile to brittle cutting mode is not possible with the Johnson-Cook flow stress model only. Instead, at least extensions, e.g. to a fracture criterion and subsequent crack propagation analysis is required. In addition, more elaborated constitutive models which consider the anisotropic nature of silicon as well as phase transformations under high compressive stresses are expected to enhance the quality of the prediction of the forces and the critical depth of cut for the ductile-to-brittle transition.

Since the temperature predictions are not satisfactory, especially for lower cut depths, the fraction of plastic work dissipated into heat (Taylor-Quinney coefficient) and the friction coefficient between silicon and diamond need to be investigated experimentally. This, together with the modelling of tool heat transfer is expected to further improve the results.

On the experimental side of the study, it was shown that, with a real grain geometry, predominantly ductile scratches with only few breakouts on the sides and bottom of the grooves are generated at depths of cut that are much larger than typically observed in cutting experiments.

## Footnotes

1 With material parameters for diamond from [7]: ${\varrho }_{Dia}=3530\frac {kg}{m^{3}}$, $cp_{Dia}=630\frac {J}{kgK}$, $\lambda _{Dia}=2200\frac {W}{mK}$
